# The crucial regulatory role of type I interferon in inflammatory diseases

**DOI:** 10.1186/s13578-023-01188-z

**Published:** 2023-12-20

**Authors:** Ling Ji, Tianle Li, Huimin Chen, Yanqi Yang, Eryi Lu, Jieying Liu, Wei Qiao, Hui Chen

**Affiliations:** 1 Faculty of Dentistry, The University of Hong Kong, Prince Philip Dental Hospital, Hong Kong, SAR People’s Republic of China; 2 Department of Stomatology, Renji Hospital, Shanghai Jiao Tong University School of Medicine, 160 Pujian Road, Shanghai, China; 3 Department of Medical Research Center, Peking Union Medical College Hospital, Peking Union Medical College, Chinese Academy of Medical Sciences, Beijing, China; 4 Division of Pediatric Dentistry and Orthodontics, Faculty of Dentistry, The University of Hong Kong, Prince Philip Dental Hospital, Hong Kong, SAR People’s Republic of China; 5 Applied Oral Sciences & Community Dental Care, Faculty of Dentistry, The University of Hong Kong, Prince Philip Dental Hospital, Level 3, 34 Hospital Road, Sai Ying Pun, Hong Kong, SAR People’s Republic of China; 6 Division of Restorative Dental Sciences, Faculty of Dentistry, The University of Hong Kong, Prince Philip Dental Hospital, Level 3, 34 Hospital Road, Sai Ying Pun, Hong Kong, SAR People’s Republic of China

**Keywords:** Type I interferon, Inflammation, Inflammatory regulation, Signaling pathways, Immune system

## Abstract

Type I interferon (IFN-I) plays crucial roles in the regulation of inflammation and it is associated with various inflammatory diseases including systemic lupus erythematosus (SLE), rheumatoid arthritis (RA), and periodontitis, impacting people's health and quality of life. It is well-established that IFN-Is affect immune responses and inflammatory factors by regulating some signaling. However, currently, there is no comprehensive overview of the crucial regulatory role of IFN-I in distinctive pathways as well as associated inflammatory diseases. This review aims to provide a narrative of the involvement of IFN-I in different signaling pathways, mainly mediating the related key factors with specific targets in the pathways and signaling cascades to influence the progression of inflammatory diseases. As such, we suggested that IFN-Is induce inflammatory regulation through the stimulation of certain factors in signaling pathways, which displays possible efficient treatment methods and provides a reference for the precise control of inflammatory diseases.

## Introduction

A significant number of individuals globally suffer from various inflammatory illnesses, such as infection, SLE, RA, systemic sclerosis (SSc), juvenile dermatomyositis (JDM), and periodontitis, presenting significant medical and socio-economic challenge [1–3]. Dysregulated IFN-Is signaling could cause inflammatory diseases, including autoimmune diseases and chronic inflections [4–6]. The regulation of IFN-I during inflammation is a complex process that typically functions as a double-edged sword, capable of inhibiting pro-inflammatory factors or triggering abnormally high levels of inflammation [7, 8].

IFN-Is belong to a class of cytokines known for their pleiotropic effects and three main functions. Firstly, IFN-Is can induce an anti-bacterial state in infected cells, controlling the spread of infectious and inflammatory agents, particularly viral pathogens [7, 9, 10]. Secondly, they regulate the innate immune response by facilitating antigen presentation and natural killer cell function [11], while mediating inflammatory pathways and cytokine [3, 12, 13]. Thirdly, IFN-Is can trigger the adaptive immune system, prompting high-affinity antigen-immune cell responses and the development of immune memory [5, 14].

Recent studies have revealed that IFN-Is also play a crucial role in the development of inflammatory diseases via regulating the associated signaling pathways [15–17]. The IFN-Is family acts as key regulatory factors for specific targets in these pathways, mediating signaling to subsequently inhibit or prompt the inflammation and immune responses [12, 18]. IFN-Is can be secreted by cytosolic receptors such as retinoic acid-inducible gene I (RIG-I), and melanoma differentiation-associated gene 5 (MDA5), and can also respond to toll-like receptors (TLRs) signaling macrophages and dendritic cells (DCs) [13, 19]. Additionally, the cytosolic GAMP synthase (cGAS) detects cytoplasmic DNA and stimulates the synthesis of circular GAMP (cGAMP), which uses the stimulator of interferon genes (STING) as a secondary receptor, and further stimulates STING-dependent inflammatory cytokines, including IFN-Is [20–22]. Subsequently, IFN-Is can bind to the heterodimeric transmembrane interferon alpha receptor (IFNAR)1 and IFNAR2, resulting in signal transducers and activators of transcription, which drive different signaling pathways through various cascades to regulate the inflammation responses [23, 24].

This review introduces the IFN-Is family involvement in the progression of inflammatory diseases and summarizes their regulatory role as crucial modulators in downstream inflammatory signaling pathways, including the Janus kinase (JAK)/signal transducers and activators of transcription (STAT) pathway, TLRs pathway, nuclear factor-κB (NF-κB) pathway, activation of the phosphoinositide 3-kinase (PI3K)/serine-threonine kinase (AKT) pathway, and mitogen-activated protein kinases (MAPK) pathway. Furthermore, the review discusses the promising potential and underlying challenges of IFN-Is-based therapy and suggests guidance to develop IFN-Is as disease-specific biomarkers and drug modulators in inflammatory diseases.

## Type I interferon

IFN-Is plays a critical role in initiating the innate immune response against a wide range of pathogens [25]. The IFN-Is family comprises IFN-α, which has 13 distinct subtypes in humans and 14 in mice, along with IFN-β, IFN-δ, IFN-ε, IFN-κ, IFN-ω, and IFN-ζ. Among these subtypes, humans can express IFN-α, IFN-β, IFN-ε, IFN-κ, and IFN-ω [26].

### The structure of interferon α/β

Among the various subtypes of type I IFNs, IFN-α and IFN-β are the most well-understood. The IFN-α protein family consists of multiple subtypes that share 76–99% amino acid identity. These subtypes contain a 23-amino-acid hydrophobic signal peptide and a 166-amino-acid mature peptide sequence. However, IFN-α2 is an exception as it encodes a 165-amino-acid protein due to a deletion at position 44. Additionally, variant polymorphic forms of IFN-α also exist, including IFN-α2a, -2b, and -2c [27].

In contrast to the IFNA genes, most mammalian genomes have not experienced duplication and expansion of the IFNB gene. Instead, these genomes contain a single gene encoding IFN-β. However, in the genomes of ruminants and pigs, evidence suggests the presence of more than one copy of the IFNB gene, indicating gene duplication in these lineages [28]. While most species have only a single IFNB gene, duplication of the IFNB gene has been observed in some members of two of the 25 Caucasian families studied [29]. Human IFN-β is a protein composed of 166 amino acids and exhibits only 25–32% sequence identity to human IFN-α proteins. In contrast, murine IFN-β is composed of 161 amino acids and shares only 19–23% sequence identity with murine IFN-α [30].

### The source of Type I interferon

Most cells in the body could produce IFN-Is in response to the stimulation of pattern recognition receptors (PRRs) by pathogens. Upon the activation by pathogens, various innate immune cells, including macrophages and DCs [31, 32], can produce IFN-Is. However, non-immune cells such as fibroblasts and epithelial cells also contribute to the production of IFN-Is [33].

PRRs are located on the cell surface, in the cytosol and endosomal compartments (Table 1), and are responsible for recognizing various pathogen-associated molecular patterns (PAMPs), including nucleic acids and non-nucleic acid PAMPs [31].

**Table 1:** Ligands and receptors induce type I interferons

Ligands	Receptor	Receptor location	References
RNA	RIG-I	Cytosol	[34]
RNA	MDA5	Cytosol	[35]
AT-rich DNA	RNA polymerase III	Cytosol	[36]
DNA	DAI	Cytosol	[37]
DNA	DEXD/H box	Cytosol	[38]
DNA	cGAS	Cytosol	[31]
Bacterial/Virus	NOD1/2	Cytosol	[39]
Bacterial	TLR 4	Cell-surface	[40]
Virus	TLR 2	Cell-surface	[41]
Double-stranded RNA	TLR 3	Endosomal	[42]
Single-stranded RNA	TLR 7/8	Endosomal	[43]
Unmethylated CpG DNA	TLR 9	Endosomal	[44]

RNA receptors RIG-I and MDA5 are the primary receptors responsible for recognizing RNA in the cytosol [31, 34, 35]. Additionally, AT-rich DNA can be transcribed by RNA polymerase III into 5′-PPP-containing RNA, which serves as a RIG-I agonist [36]. Other DNA motifs in the cytosol can be recognized by various receptors, including DNA-dependent activator of IFN-regulatory factors (DAI), DEAD and DEAH box (DEXD/H box) helicases, and cGAS [31, 32, 37, 38], all of which are highly associated with the induction of IFN-Is. Furthermore, the cytosolic molecular sensors NOD-containing protein 1 (NOD1) and NOD2 are specialized in recognizing bacteria and viruses, leading to IFN-Is production [39].

In addition to cytosolic receptors, TLRs also play a role in activating pathways that lead to IFN-Is production. Among cell-surface TLRs, TLR 4 recognizes lipopolysaccharide (LPS) from bacteria and induces IFNβ through Toll-receptor-domain-containing adapter-inducing interferon-β (TRIF)-dependent pathway [40]. In contrast, other cell-surface TLRs signaling is responsible for IFN-Is production in response to viruses through myeloid differentiation primary response 88 (MyD88)-dependent pathway [41].

Endosomal compartments are also involved in IFN-Is production, with TLR 3, TLR 7, TLR 8, and TLR 9 being responsible for recognizing different types of PAMPs to induce IFN-Is. TLR 3 responds to double-stranded RNA [42], while TLR 7 and TLR 8 recognize single-stranded RNA [43]. TLR 9 responds to unmethylated CpG DNA [44].

## Type I interferon signaling in inflammation

**Fig. 1:**
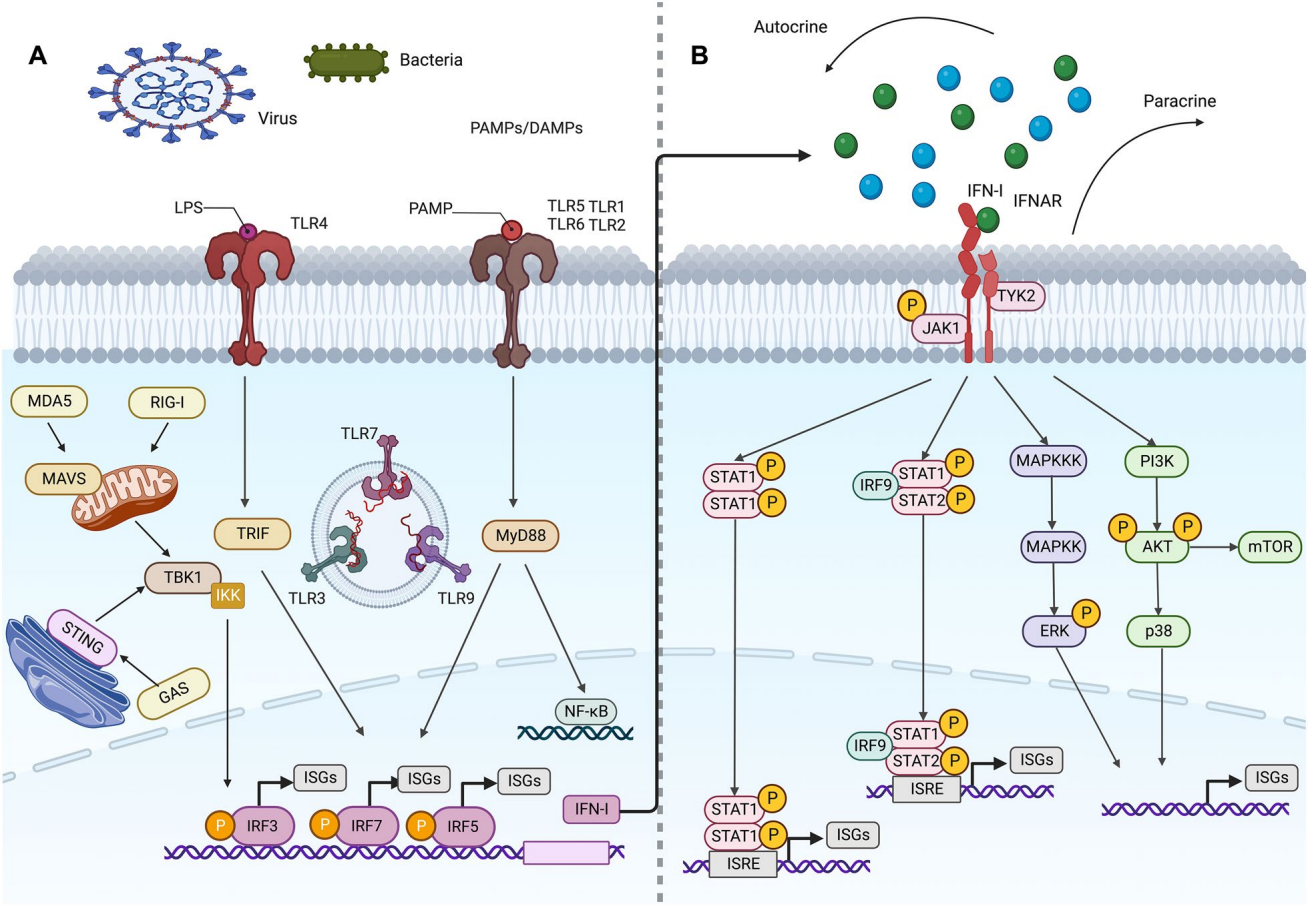
Different signaling pathways are involved in the inflammatory regulation of IFN-I. **A** After being stimulated by bacteria, viruses, PAMP/DAMP, etc. in the external environment, the DNA sensors activate STING, which moves to the Golgi and is phosphorylated by TBK1, allowing for the phosphorylation and activation of IRF 3. Upon binding to their ligands, RIG-I and MDA5 engage MAVS, leading to activation of TBK1 and members of the IKK family of kinases. Similarly, TLRs signal through MyD88 and TRIF adaptor molecules, leading to the activation of TBK1 and members of the IKK family. These kinases trigger the phosphorylation, activation, and dimerization of IRFs and the release of NF-κB. IRFs and NF-κB then migrate into the nucleus where they bind to promoter regions of IFN-I and other target genes, thereby stimulating IFN-I as well as anti-inflammatory and pro-inflammatory cytokine gene transcription. **B** In the canonical IFN-I signaling pathway, IFN-I binding with IFNAR results in the phosphorylation of JAK1 and TYK2, which then recruit and activate STAT proteins, leading to their trimerization or dimerization and nuclear translocation. Two distinct transcriptional complexes are formed, which regulate the expression of different ISGs' in a sequence-dependent manner. ISGF3, a trimerized complex formed by STAT1, STAT2, and IRF 9, recognizes the ISRE motif and induces a group of gene expression. The other complex formed by STAT1 homodimers binds to the GAS motif and mainly active inflammatory gene expression. B In the uncanonical IFN-I signaling pathway, IFN-I can also induce a set of genes expression independent of STATs, such as MAPKs and PI3K pathways. Additionally, IFN regulates some ISGs’ translation through the mTOR signaling pathway

The regulation of IFN-Is in inflammation is a complex process that involves inducing cell-intrinsic antimicrobial states to limit the spread of infectious agents, modulating innate immune responses to inhibit cytokine production, and activating the adaptive immune system, which can lead to either restrained pro-inflammatory pathway or excessive abnormal inflammation [7, 8]. This process is controlled by multiple critical pathways. There is compelling evidence that the IFN-I family serves as mediators for their specific targets in these pathways to regulate cascade reactions, thus subsequently suppressing or promoting sustained inflammation as well as immune activation [45, 46]. This review summarizes IFN-Is that control specific factors to promote or inhibit inflammation through mediating downstream signaling pathways, including the JAK/ STAT pathway, TLRs pathway, NF-κB pathway, PI3K/AKT pathway, and MAPK pathway. (Fig. 1).

### JAK/STAT pathway

After production, IFN-Is activate a wide range of gene transcription in an autocrine and paracrine manner by triggering the downstream signals [47]. The cognate receptor complex of IFN-Is consists of the ubiquitously expressed transmembrane IFNAR1 and IFNAR2, which initiates signaling cascades upon binding [12, 48]. JAK1 and non-receptor tyrosine kinase 2 (TYK2) are phosphorylated and activated by IFNAR [24], subsequently inducing phosphorylation and dimerization of transcription factors STAT1 and STAT2 [49, 50]. The heterodimer then translocated to the nucleus and recruits IFN-regulatory factor (IRF) 9 to form STAT1-STAT2-IRF 9 tri-complex (IFN-stimulated gene factor 3, ISGF3) [51, 52]. The complex binds to IFN-stimulated response elements (ISRE), a DNA sequence motif, to activate the transcription of a group of genes known as IFN-stimulated genes (ISG) [53, 54]. By regulating IFN-α and IFN-β signaling, ISGs control inflammation. Additionally, IFN-I could also induce STAT1 to form homodimers that are not assembled with IRF 9, subsequently binding to a unique consensus sequence in the ISG promoter called the gamma-activating sequence (GAS) [55]. (Table 2).

**Table 2:** The inflammatory regulation of IFN-I signaling in the JAK/STAT pathway

Study	Types of IFN-I	Target of action	Stimuli	Experimental models	Effect	Diseases
Dean et al. 2021 [56]	IFN-I	IFN-I	COVID-19	Peripheral blood of COVID-19 patients	Duality	Viral infection
Nocito et al. 2020 [57]	IFN-α	IFN-α	Captopril	MRL/lpr and MRL/wt female mice	Anti-inflammatory	SLE
Kitamura et al. 2020 [58]	IFN-α	IFN-α	Experimental radiation	C57BL/6 mice	Pro-inflammatory	Esophagitis
Fernandez-Sendin et al. 2020 [59]	IFN-α	IFN-α	Apolipoprotein AI mimetic peptide L37pA	C57BL/6 mice	Anti-inflammatory	Viral infection
Chen et al. 2022 [60]	IFN-α	IFN-α	A. cinnamomea	BHK-21 cells	Anti-inflammatory	Viral infection
Zhang et al. 2022 [61]	IFN-β	IFN-β	PON1	PAM	Pro-inflammatory	Viral infection
Li et al. 2020 [62]	IFN-β	IFN-β	MiR-30a	HeLa cells and 293 T cells	Pro-inflammatory	Viral infection
Jang et al. 2018 [63]	IFN-β	IFN-β	Enhanced IFN-β	Human tissue from a patient with sinusitis	Pro-inflammatory	Sinusitis
Cook et al. 2019 [64]	IFN-α and IFN-β	IFN-α and IFN-β	CHIKV	C57BL/6 mice	Anti-inflammatory	Viral infection
Lei et al. 2021 [65]	IFN-α and IFN-β	IFN-β	NRF2	mtDNA mutant mice	Pro-inflammatory	Not given
Arimori et al. 2013 [66]	IFN-α and IFN-β	IFNAR	IFNAR KO	IFNAR KO mouse models	Pro-inflammatory	Viral infection
Sontheimer et al. 2017 [67]	IFN-α and IFN-β	IFNAR	UVB and IFNAR KO	IFNAR KO mouse models	Pro-inflammatory	SLE
Ansar et al. 2021 [68]	IFN-α	IFNAR	RS	BAL from mice	Anti-inflammatory	Viral infection
Cagliani et al. 2019 [69]	IFN-α	IFNAR	IFNAR1-Ab	C57BL6 mice	Anti-inflammatory	HS
D'Souza et al. 2021 [70]	IFN-α and IFN-β	IFNAR	MO-IFN	C57BL/6 mice	Pro-inflammatory	Not given
Chan et al. 2020 [71]	IFN-α and IFN-β	IFNAR	Activation of IFN/ IFNAR axis	Mouse primary adipocytes	Pro-inflammatory	Obesity
Minayoshi et al. 2018 [72]	IFN-α and IFN-β	IFNAR	Man-HSA(D494N)-IFNα2b	C57BL/6 mice	Anti-inflammatory	Hepatitis
Tanaka et al. 2012 [73]	IFN-α and IFN-β	STAT1	Damaged STAT1	Gingival tissue samples	Pro-inflammatory	Periodontitis
Nataraja et al. 2022 [74]	IFN-α	STAT1	GILZ	C57BL/6 mice	Anti-inflammatory	SLE
Chen et al. 2022 [75]	IFN-α	STAT1	Menthone	CIA mouse models	Anti-inflammatory	RA
Hu et al. 2022 [76]	IFN-β	STAT1	RUNX1	A549 cells	Anti-inflammatory	Viral infection
Yang et al. 2020 [77]	IFN-α and IFN-β	STAT1	hsa_circ_0060450	Peripheral blood of T1DM patients and healthy controls	Anti-inflammatory	T1DM
Gothe et al. 2022 [78]	IFN-α	STAT2	STAT2 deficiency	pluripotent stem cell-derived macrophages	Pro-inflammatory	Not given
Wilson et al. 2019 [79]	IFN-α and IFN-β	STAT2	S. typhimurium	C57BL/6 mice	Pro-inflammatory	Bacterial infection
Shen et al. 2020 [80]	IFN-β	STAT2	PCV3 Cap	HEK 293 T and PK15 cells	Pro-inflammatory	Viral infection
Kozela et al. 2010 [81]	IFN-β	STAT3	CBD	BV-2 murine microglial cell line	Anti-inflammatory	Not given
Racicot et al. 2016 [82]	IFN-β	STAT3	Upregulated STAT3	Human trophoblasts SW.71 and C57BL/6 mice	Pro-inflammatory	Placental inflammation
Febvre-James et al. 2018 [83]	IFN-β	JAK	Ruxolitinib	PBMCs	Anti-inflammatory	Not given
Klopfenstein et al. 2021 [84]	IFN-β	JAK and STAT	MRSA	C57BL/6 mice	Pro-inflammatory	Bacterial infection
Hadjadj et al. 2020 [85]	IFN-α and IFN-β	JAK and STAT1	COVID-19	sera from COVID-19 patients	Anti-inflammatory	Viral infection
Stefan et al. 2017 [86]	IFN-α and IFN-β	IL-10R	S. typhimurium	C57BL/6 mice	Pro-inflammatory	Salmonella colitis
Qu et al. 2015 [87]	IFN-α and IFN-β	MCPIP-1 and miR-146a	IFN-α and IFN-β	THP-1 cells	Pro-inflammatory	SLE
Zhang et al. 2015 [88]	IFN-α and IFN-β	ISG15	USP18	Human blood sample cells	Pro-inflammatory	Not given

#### Gene activation triggered by IFN-Is

Activation of IFN-I occurs through JAK/STAT signaling pathway, and its role is two-fold, with both pro-inflammatory and anti-inflammatory effect [2, 89]. For instance, research on COVID-19 demonstrated that asymptomatic patients developed a protective IFN-I inflammatory response, whereas severe COVID-19 patients had increased expression of ISGs and excessive inflammation reaction [56]. The angiotensin-converting enzyme inhibitor captopril has been found to reduce circulating and tissue IFN-α levels, along with decreased inflammation of peripheral and central nervous system in lupus-prone mice [57]. Similarly, in a study conducted by Kitamura et al., it was observed that radiation up-regulated the gene expression level of IFN-α in esophageal tissue. It should also be noted that anti-IFN-α neutralizing antibody improved radiation-induced esophageal mucosal inflammation, while IFN-α receptor agonist (RO8191) had the opposite effect, reflecting the pro-inflammatory properties of IFN-α [58]. Additionally, co-expression of the apolipoprotein AI mimetic peptide L37pA with IFN-α resulted in a significant reduction of IFN-α expression, thereby inhibiting inflammatory pathways and responses related to PAMPs and immune cells. This suggests a possible effective treatment for inflammatory processes [59]. Conversely, Antrodia camphorata (A. cinnamomea) was observed to increase the level of IFN-α after dengue virus (DENV) infection, playing an antiviral and anti-inflammatory role [60].

Upon viral infection, IFN-β plays a crucial role. However, under porcine reproductive and respiratory syndrome virus (PRRSV) infection, Paraoxonase-1 (PON1) has been found to inhibit the IFN-β pathway to promote PRRSV replication by interacting with PRRSV nonstructural protein 9 (Nsp9), resulting in an expansion of infection and inflammation [61]. Besides, microRNA (miR)-30a has been identified as a potent negative regulator of IFN-β signaling. It suppresses tripartite motif protein 25 (TRIM25) expression and TRIM25-mediated RIG-I ubiquitination to suppress IFN-β activation and production, leading to enhanced CVB3 replication and inflammation [62]. While IFN-β has been known for its antiviral function, its enhanced response has been found to lead to eosinophilic chronic rhinosinusitis via C–C Motif Chemokine Ligand 11(CCL11) [63]. Interestingly, Cook et al. reported that during acute chikungunya virus (CHIKV) infection, both IFN-α and IFN-β play protective roles, but through different mechanisms: IFN-α restricts CHIKV replication and spread, while IFN-β limits neutrophil-mediated inflammation to prevent CHIKV pathogenesis [64].

#### IFNAR

IFNARs are essential for the cascade reaction initiated by IFN-I signaling. Inhibition or blocking of causes significant changes in the downstream pro-inflammatory factors and the inflammatory environment [3, 90]. It has been reported that interleukin (IL)-10 levels are significantly reduced in IFNAR knockout (KO) mice during influenza virus infection. The antiviral and anti-inflammatory activities of IFN-I are abolished [66]. In contrast, ultraviolet B (UVB)-irradiated IFNAR KO mice displayed elevated levels of pro-inflammatory cytokines and more severe histological inflammation, suggesting the protective effects of IFN-I [67]. However, IFN-α acts differently under respiratory syncytial virus (RSV) infection. RSV-infected IFNAR-deficient mice showed decreased IFN-α production but demonstrated significantly reduced secretion of pro-inflammatory cytokines and chemokines in the airways. This suggest that IFN-I may contribute to RSV induced inflammation [68]. Studies have shown that using IFNAR1-Ab to bind IFNAR achieves a therapeutic effect by reducing the protein levels of pro-inflammatory cytokines and relieving inflammation and tissue damage [69]. Moreover, scRNA-seq has identified a novel IFN-I signaling-dependent monocyte subpopulation (MO-IFN) that upregulates IFNAR1 expression to increase IFN-I, thereby contributing to monocyte infiltration and the increased inflammation base level [70]. Additionally, Chan et al. has found that activation of the IFN/IFNAR axis increases pro-inflammatory cytokine levels in adipocytes, suggesting further investigation is necessary to understand the roles of adipocyte inflammation in disease pathogenesis [71].

#### STAT

STAT is a family of transcription factors related to signal transduction and transcriptional activation, which mediates many aspects of cellular immunity and has been identified to significantly regulate IFN-Is signaling [91]. In detail, STAT could combine with IRF to form a complex, and it subsequently binds to the ISRE promoter to induce ISG expression, thereby affecting the regulation of inflammatory factors [49].

In patients with periodontitis, reduced expression of the STAT1 gene leads to impaired downstream of IFN-I signaling, contributing to decreased IFN-I activation and excessive periodontal inflammation [73]. In patients with SLE, glucocorticoid-induced leucine zipper (GILZ) gene directly binds to STAT1, blocking its nuclear translocation and reducing IFN-α-induced gene expression, thereby blocking the pro-inflammatory response of IFN-α [74]. In addition, menthone promotes K48-linked polyubiquitination of TKY2, indirectly inhibiting STAT1 instead of inducing its phosphorylation, significantly restraining local inflammation in collagen II-induced arthritis (CIA) mice [75]. However, during influenza A virus (IAV) infection, STAT1 expression could be hindered by RUNX1, a transcription factor, which subsequently attenuates IFN-β signaling, promoting the expansion of infection and inflammation [76]. Furthermore, hsa_circ_0060450, a circular RNA, functions as a sponge for miR-199a-5p to release its target gene, src homology 2-containing protein tyrosine phosphatase 2 (SHP2), which further targets the inhibition of STAT1, blocking the activation of IFN-I and inhibiting macrophage-mediated inflammation. [77].

STAT2 deficiency may cause failure of feedback from IFN-α signaling, leading to immune dysregulation. Aberrant IFN-α signaling can also switch transcriptional output into a clinically evident inflammatory response [78]. STAT2-dependent IFN-I signaling could accelerate an inflammatory environment due to its release of inflammatory factors by disrupting hypoxia during pathogenic Salmonella typhimurium infection [79]. Additionally, Shen et al. [80] discovered that the capsid protein (Cap) of porcine circovirus 3 (PCV3) could interact with the transactivation domain of STAT2, hindering the the expression of IFN-β and preventing the defense against viral infection and inflammation by binding to ISRE and prevent the ISRE of IRF 9-S2C.

STAT3 indirectly regulates the inflammatory response related to IFN-Is mainly through STAT1 and STAT2. Cannabidiol (CBD) has been found to be able to inhibit Socs3, the main negative regulator gene of STAT3, and downregulated STAT3 blocks the activation of STAT1 transcription factor, inhibiting the IFN-β-dependent pro-inflammatory process [81]. This conclusion was further partially supported by another study, which showed a positive correlation between the expression of STAT3 phosphorylation and IFN-β: the decrease in STAT3 expression suppressed the IFN-β pathway, but resulted in a significant increase in inflammatory cytokines [82].

### Toll-like receptor pathway

The canonical IFN-I-JAK/STAT signaling pathway does not operate independently but engages in extensive and critical communication and crosstalk with other signaling pathways, such as PRRs, including TLRs [3, 35]. Downstream of the signaling pathways of host germline-encoded PRRs, which are expressed on the cell membrane or in the cytoplasm of the cells of the innate immune system, IFN-Is can be produced in response to PAMP that includes pathogenic nucleic acids, LPS, and proteins, or in response to host damage-associated molecular patterns (DAMP) [5, 90]. After intracellular TLRs (TLR 3, TLR 7/8, and TLR 9) are activated, IFN-I production is subsequently induced by IRF 3, IRF 7, and IRF 5 [92]. TLRs signaling can be broadly classified into two pathways: the MyD88-dependent and the TRIF-dependent pathway [93]. While other TLRs can activate through the MyD88-dependent pathway [94], only TLR 3 and TLR 4 activate through the TRIF-dependent pathway [95] (Table 3).

**Table 3:** The inflammatory regulation of IFN-I signaling in the Toll-like receptor pathway

Study	Types of IFN-I	Target of action	Stimuli	Experimental models	Effect	Diseases
Veenhuis et al. 2017 [96]	IFN-α and IFN-β	TLR 7	HIV	Serum samples from HIV patients	Anti-inflammatory	HIV
Yang et al. 2016 [97]	IFN-β	TLR 3 and TLR 7	Enteric viruses	BALB/c mice	Anti-inflammatory	Colitis
Sekheri et al. 2022 [98]	IFN-β	TLR 9	ALX/FPR2	Human neutrophils	Anti-inflammatory	ARDS
Allen et al. 2021 [99]	IFN-α	TLR 7	HIV	PBMCs from SLE patients	Anti-inflammatory	SLE
Chang MY et al. 2017 [100]	IFN-β	TLR 3 or TLR 4	LPS and versican	Transgenic mice	Anti-inflammatory	Bacterial infection
Dhariwala et al. 2017 [101]	IFN-β	TLR 7	Yersinia pestis	C57BL/6 mice	Anti-inflammatory	Bacterial infection
Auger et al. 2017 [102]	IFN-β	IRF 3 and IRF 7	Streptococcus suis Serotype 2	C57BL/6 mice	Duality	Bacterial infection
Cordoba-David et al. 2022 [103]	IFN-α and IFN-β	IRF 3	LPS	mouse MCT cells	Anti-inflammatory	Nephritis
Artusa et al. 2022 [104]	IFN-β	IRF 3	Coffee extracts	THP-1 cells	Anti-inflammatory	Not given
Zhou et al. 2020 [105]	IFN-β	IRF 3	GTD	C57BL/6 mice	Anti-inflammatory	Viral infection
Fritsch et al. 2022 [106]	IFN-β	IRF 3	CCI or sham injury	C57BL/6 mice	Pro-inflammatory	TBI
Huang et al. 2022 [107]	IFN-β	IRF 3	Polβ	MEFs	Pro-inflammatory	Cancer
Onsa-Ard et al. 2022 [108]	IFN-β	IRF 3	RRBE	RAW 264.7 macrophages	Anti-inflammatory	Not given
Liu et al. 2022 [109]	IFN-β	IRF 3	RNF 5	Human corneal epithelial cells	Pro-inflammatory	Viral infection
Wu et al. 2019 [110]	IFN-α and IFN-β	IRF 7	IRF 7 KO	C57BL/6 mice and skin tissue from a patient with SSc	Pro-inflammatory	SSc
He et al. 2019 [111]	IFN-α and IFN-β	IRF 7	Papain or IL-33	C57BL/6 mice	Pro-inflammatory	Allergic airway inflammation
Zhou et al. 2015 [112]	IFN-α and IFN-β	IRF 7	AIP	HEK293T cells	Pro-inflammatory	Viral infection
Hu et al. 2018 [113]	IFN-α and IFN-β	IRF 7	High glucose	THP-1 cells	Pro-inflammatory	**Diabetes**
Ren et al. 2016 [114]	IFN-α and IFN-β	IRF 7	LPS or virus	IFNAR ± mice	Anti-inflammatory	Viral infection
Trevejo-Nunez et al. 2021 [115]	IFN-α and IFN-β	IRF 7	Regnase-1	C57BL/6 mice	Anti-inflammatory	Bacterial infection
Valaperti et al. 2014 [116]	IFN-α and IFN-β	RIG-I	CAP	CAP (−/−) mice	Anti-inflammatory	Myocarditis
Villamayor et al. 2023 [117]	IFN-α	RIG-I	IFI 6	293 T and MDCK cells	Pro-inflammatory	Viral infection
Simpson et al. 2017 [118]	IFN-α and IFN-β	RIG-I and RLR	IPS-1	C57BL/6 mice	Anti-inflammatory	Viral infection
Zheng et al. 2022 [119]	IFN-β	RIG-I-MAVS	NSP5 and N proteins of SARS-CoV-2	HEK‐293 T, HeLa, and Vero E6 cells	Pro-inflammatory	Viral infection
Han et al. 2021 [120]	IFN-β	RIG-I/MDA5-MAVS	SARS-CoV-2 ORF9b	HEK‐293 T, HeLa, and Vero E6 cells	Pro-inflammatory	Viral infection
Zheng et al. 2020 [121]	IFN-β	RIG-I/MDA5-MAVS	SARS-CoV-2 M protein	HEK293, HEK293T, HeLa, and Vero cells	Pro-inflammatory	Viral infection
Deng et al. 2023 [122]	IFN-β	RIG-I/MDA5-MAVS	SARS-CoV-2 NSP7	HEK293T, HeLa, Vero, and HK-2 cells	Pro-inflammatory	Viral infection
Deng et al. 2023 [123]	IFN-β	RIG-I/ MDA5-MAVS	SARS-CoV-2 NSP8	HEK293T, HeLa, Vero, HK-2, and L929 cells	Pro-inflammatory	Viral infection
Liu et al. 2018 [124]	IFN-β	MAVS	TRIM21	BALB/c mice	Anti-inflammatory	Viral infection
Gutierrez-Merino et al. 2020 [125]	IFN-α and IFN-β	MAVS	LAB	BMDMs, PBMCs and THP-1 cells	Anti-inflammatory	Bacterial infection
Killarney et al. 2023 [126]	IFN-β	MAVS	Chemotherapy	A375 and Colo205 cells	Anti-inflammatory	Not given
Huang et al. 2022 [127]	IFN-β	MAVS	Sorafenib	293 T cells	Anti-inflammatory	Not given
Han et al. 2022 [128]	IFN-β	MAVS	RNF114	BALB/c mice	Pro-inflammatory	Viral infection
Pons et al. 2021 [129]	IFN-α and IFN-β	cGAS	CDT	HeLa cells and mouse embryonic fibroblasts	Anti-inflammatory	Viral infection
Hsin et al. 2021 [130]	IFN-β	STING	Hepsin	human hepatocytes and HEK293T cells	Pro-inflammatory	Viral infection
Fischer et al. 2020 [131]	IFN-α and IFN-β	cGAS/STING	Cutibacterium	THP-1 cells	Anti-inflammatory	Bacterial infection
Vail et al. 2021 [132]	IFN-β	cGAS/STING	Rhodococcus equi	BMDMs	Pro-inflammatory	Bacterial infection
Wang et al. 2022 [133]	IFN-α and IFN-β	RIPK1-TBK1	Caspase 8	C57BL/6 mice	Anti-inflammatory	Viral infection
Torre et al. 2017 [134]	IFN-α and IFN-β	TRIM 25	USP 15	C57BL/6 mice	Anti-inflammatory	Neuroinflammation
Li et al. 2022 [135]	IFN-α	SLC15A4	miR-31-5p	Peripheral blood sample from SLE patients	Anti-inflammatory	SLE

#### TLR

As discussed previously, the activation of TLRs can affect the production of IFN-Is and their interaction with the JAK/STAT signaling pathway, thereby influencing the occurrence and progression of inflammation [136, 137]. TLR 7 signaling has been identified as a prerequisite for human immunodeficiency virus (HIV)-induced IFN-Is production, and antibodies produced during untreated HIV infection may contribute to the sustained high-level IFN-Is response during the infection, suggesting a new mechanism of immune activation through TLRs [96]. Moreover, Yang et al. have demonstrated that upon virus recognition, TLR 3 and TLR 7 activation leads to IFN-β production, which can improve inflammation progression, displaying a protective role in inflammatory regulation [97]. In the MyD88-dependent pathway of TLRs signaling, blocking TLR activation has been considered a potential strategy for addressing excessive inflammation mediated by IFN-Is [98]. For instance, chloroquine loaded by filamentous micelles (CQ-FM), a TLR antagonist, can inhibit TLR activation, leading to a significant reduction in downstream IFN-Is production and decreased inflammation [99]. The TRIF-dependent pathway typically regulates IFN-Is production during bacterial infection. Upon being stimulated by IFN-stimulated genes, such as versican, TLR 3 or TLR 4 could be triggered via LPS to activate the signaling cascade of TRIF adapter, IFN-I as well as IFNAR, allowing IFN-Is to fully exert their anti-inflammatory properties [100]. Interestingly, the current research has found that during Yersinia pestis infection, TLR 7 might have an unconventional signal transduction adapter independent of MyD88, which induces IFN-Is production, inhibiting inflammation caused by the plague [101].

#### IRF

The IRF family of transcription factors plays a crucial role in IFN-IS induction, with IRF 3 and IRF 7 acting as major mediators downstream of cytoplasmic RNA and DNA receptors, as well as TLRs pathways [138–140]. Auger et al. have found that during *Streptococcus suis* infection, TLR 7 and TLR 9 could recognize bacterial nucleic acids, leading to the activation of IRF 3 and IRF 7, which then induce IFN-β production. The IFN-Is participate in modulating systemic inflammation in host defense, displaying an anti-inflammatory role, when induced relatively mild virulent strains. However, highly virulent strains rapidly induce septic shock and inflammation, which is abnormally regulated by IFN-Is [102].

IRF 3 is expressed ubiquitously and can be activated through phosphorylation to facilitate dimerization, nuclear translocation, the combination with the co-activator cAMP-response element binding protein (CREB)-binding protein (CBP), subsequently binding to canonical ISRE in the promoter of IFN-β and IFN-α [141–144]. Studies have shown that after the combination of LPS and TLR 4, IRF 3 can be activated via phosphorylation of kinases TANK-binding kinase 1 (TBK1) and inhibitor of NF-κB (IκB) kinase (IKKε), inducing ISG to produce IFN-Is, modulating the process of inflammation [103]. In a separate study, Artusa et al. reported that green coffee and roasted coffee extract can inhibit the effect of IRF 3, thereby inhibiting excessive IFN-β-induced inflammation [104]. Conversely, during viral infection, gastrodin (GTD) can promote the activation of IRF 3 in macrophages to facilitate the production of IFN-Is, resisting inflammation and anti-viral infection [105]. Another significant pathway for the production of IFN-Is through IRF 3 is cGAS/ STING signaling. When cGAS binds to double-stranded DNA (dsDNA), it can be activated and convert adenosine 5'-tri Phosphate (ATP) and guanosine 5'-triphosphate (GTP) to cGAMP, which together with other cyclic dinucleotides (CDNs) signal to STING downstream in the endoplasmic reticulum (ER), subsequently activating IRF 3 in the nucleus, leading to secretion of IFN-Is [145, 146]. Mitochondria can release DNA into the cytoplasm, binding cGAS and promoting the activation of STING, which further activates IRF 3 through phosphorylation by TBK1, contributing to the increased concentrations of IFN-Is as well as inflammatory cytokines in the innate immune response, facilitating the progression of inflammation [106, 147]. Meanwhile, the leaked mitochondrial DNA (mtDNA) could be recognized by TLR9 and trigger MyD88-dependent signaling, promoting pro-inflammatory cytokine expression such as tumor necrosis factor (TNF) as well as IL and IFN-Is secretion through ISG upregulation [148, 149]. This conclusion has been confirmed by demonstrating that oxidized mtDNA drove IFN-Is secretion through the TLR9 pathway in humans with SLE [150, 151]. It is worth noting that during this process, oxidized mtDNA driven by TLR signaling activates the nucleotide-binding oligomerization domain, leucine-rich repeat and pyrin domain-containing 3 (NLRP3) inflammasome, which in turn facilitates IL-1β maturation in this process, crucially participating in the activation as well as regulation of inflammation [152]. Apart from that, it is currently found that inhibiting mtDNA synthesis through IRF ablation could prevent NLRP3 inflammasome activation and suppress this process of inflammation [153, 154]. DNA polymerase β (Polβ) deficiency can also result in the accumulation of DNA damage in the cell and trigger the leakage of damaged DNA into the cytoplasm, activating STING and facilitating the IRF 3 signaling cascade, promoting the activation of TBK1-phosphorylated IRF 3 to translocate into the nucleus, enhancing the expression of IFN-Is and pro-inflammatory cytokines [107]. Several factors can modulate the level of IFN-Is through IRF 3 via distinct ways, regulating the innate immune response and inhibiting pro-inflammatory signaling. For instance, red rice bran extract (RRBE) can inhibit the phosphorylation of STING, blocking the activation of IRF 3 to hinder initiation of IFN-Is signaling, which function as pro-inflammatory cytokines [108]. Additionally, the E3 ligase RNF 5 can also limit the signaling of IRF 3 through targeting STING, suppressing the production of IFN-Is, which instead promotes viral replication and abnormal inflammation development [109].

In contrast to IRF 3, IRF 7 is usually expressed at very low levels, except in plasmacytoid DCs (pDCs) [155, 156]. IRF 7 can be activated by phosphorylation of TBK1/IKKε and TRIF-dependent pathways downstream of cytoplasmic RNA/DNA sensors, leading to its entry into the nucleus to dimerize with IRF 3, transcriptionally activating and inducing the expression of IFN-α and IFN-β [157, 158]. In addition, IRF-7 is essential for pathways involving MyD88 recruitment, leading to IKKα activation and driving IFN-α and IFN-β expression in response to viruses [159–161]. Furthermore, IRF 7 can form a feed-forward loop with IFN-Is, maximizing the expression of IFN-Is and continuously producing a large number of IFN-Is, acting as a positive regulator of IFN-Is [162, 163]. However, IRF7 can also facilitate inflammation and the progress of inflammatory diseases. For instance, gene and protein levels of IRF 7 were significantly enhanced in skin and cultured fibroblasts from patients with SSc, and IRF 7 knockout mice exhibited lower levels of pro-fibrotic factors and less inflammatory response [110]. Additionally, asthmatic patients with higher levels of type 2 innate lymphoid cells (ILC2) in peripheral blood and bronchoalveolar lavage fluid (BALF) to drive inflammation compared had greater IRF 7 expressions in murine lung ILC2s after t stimulation from papain or IL-33 [111]. Furthermore, aryl hydrocarbon receptor-interacting protein (AIP) can inhibit IRF 7 by antagonizing the nuclear localization of IRF 7, hindering the production of IFN-Is induced by IRF 7, reducing the immune response and promotes abnormal inflammation [112]. Nevertheless, IRF7 can also restrain inflammation and suppress the progress of inflammatory diseases in certain conditions. A study has shown that when USP25 was upregulated by virus infection or LPS, IRF 7 could directly bind to two conserved IRF binding sites on the USP25 promoter, driving the transcription of USP25 and promoting the secretion of IFN-Is to adjust the innate immune signal transduction and exhibit an anti-inflammatory effect [114].

#### RIG-I

Host cells sense invading viruses as well as launch innate immune responses to resist infection, in which detection of viral nucleic acids via RIG-I could produce activated signaling, leading ultimately to the secretion of IFN-Is [164]. In this process, the protein activator of the interferon-induced protein kinase (PACT), also referred to as the protein kinase, interferon-inducible double-stranded RNA-dependent activator (PRKRA), is a crucial component in initiating and maintaining RIG-I-dependent antiviral responses [165]. PACT physically binds to the C-terminal repression domain of RIG-I and then enhances the activation of RIG-I through poly (I:C) of intermediate length [166, 167]. Afterward, RIG-I functions as a virus sensor that triggers the innate antiviral response and could be activated by dsRNA [168]. Then, its N-terminal caspase activation and recruitment domain (CARD) will migrate and link to the CARD on the mitochondrial antiviral signaling protein (MAVS), activating the signal transduction of IFN-Is, and subsequently promoting the innate immune response including inflammatory response [169–171]. It is noteworthy that cytoplasmic RIG-I can upregulate the secretion of IRF 3-dependent IFN-Is and reduce the level of MDA5 via combining with c-Cbl-associated protein (CAP), reducing cytotoxicity and alleviating myocarditis [116]. In contrast, Villamayor et al. have revealed a novel interaction between RIG-I and IFN-α-inducible protein 6 (IFI6), which affects RIG-I activation through mediating RNA binding, resulting in negative regulation of innate immunity and excessive inflammation [117]. Moreover, triggered by IFN-Is promoter stimulator-1 (IPS-1) signaling, RIG-I-like receptors (RLRs) could collaborate with TLR 7 to advance pDC recruitment and IFN-α production, restraining the host response to pneumonia viral infection and thus preventing viral bronchiolitis [118]. Apart from that, interestingly, during severe acute respiratory syndrome coronavirus 2 (SARS-CoV-2) infection, RIG-I-MAVS siganling could also be blocked by the NSP5 and N proteins of SARS-CoV-2, which could inhibit RIG-I-induced IFN-Is response, resulting in weakening antiviral immunity [119].

#### MAVS

The protein MAVS is a crucial component of innate immunity, functioning as a central pivot for signal transduction initiated by TLR, RIG-I-like receptors, and MDA5 [172]. Notably, PACT, which is linked to the host antiviral response, promotes the formation of RNA-induced MDA5 oligomers in this process, thereby being beneficial to the initiation of the IFN-Is signaling cascade associated with MAVS [173, 174]. Additionally, MAVS can stimulate the IFN-beta promoter by activating IRF 3, modulating inflammatory signaling related to IFN-Is [172, 175, 176]. However, during infection, a range of factors that target MAVS to affect the control of inflammation by IFN-Is. For instance, TRIM21, a regulator of tissue inflammation and pro-inflammatory cytokine production, interacts with MAVS during coxsackievirus B3 (CVB3) infection to promote upregulation of IFN-β signaling, enhancing host defense against inflammation [124]. Similarly, under the influence of lactic acid bacteria Lactobacillus (LAB), MAVS activates the production of IFN-Is, but directs bacteria-specific immunity [125]. In chemotherapy, mitochondrial RNA (mtRNA) induced by apoptosis damage could activate MDA5, which subsequently upregulates MAVS to promote IFN-Is signaling, suppressing the inflammation caused by the cytoplasmic release of mtRNA in the presence of caspases inhibition [126]. Nevertheless, instead of activating MAVS, sorafenib mainly limits the recruitment of MAVS to negatively regulate IFN-Is signaling. The inhibition of excessive inflammation can prevent the occurrence of inflammatory diseases [127]. Conversely, RING finger protein 114 (RNF114), an E3 ubiquitin ligase, can restrain the production of IFN-Is via interacting with MAVS and inhibiting RIG-I-mediated signaling, promoting viral replication and excessive inflammation [128].

Furthermore, it is worth noting that SARS-CoV-2 could cause coronavirus disease 2019 (COVID-19), and has a non-negligible correlation with RIG-I, MAVS, and MDA5 as well as their involvement in IFN-Is signaling in this process. Han et al. reported that SARS-CoV-2 ORF9b suppressed the components of the cytoplasmic dsRNA sensing pathway transduced via RIG-I/MDA5-MAVS signaling to antagonize the induced activation of IFN-Is, leading to the development of infection and inflammation [120]. Besides, the SARS-CoV-2 membrane (M) protein has also been shown to display a similar role in infections caused by SARS-CoV-2 [121]. Moreover, recent research has confirmed that both SARS-CoV-2 NSP7 and SARS-CoV-2 NSP8 could prevent the formation of the RIG-I/MDA5-MAVS signal body, thereby restraining the induction of IFN-Is, and then facilitating the generation of inflammation as well as virus replication [122, 123].

### NF-κB pathway

NF-κB is a group of proteins that function as dimerizing transcription factors to regulate gene expression and various biological processes, including innate and adaptive immunity, as well as inflammation [177]. NF-κB/Rel proteins bind to the inhibitor of NF-κB (IκB) proteins and are thereby inhibited. However, the activation of proinflammatory cytokines, LPS, growth factors, and antigen receptors stimulate an IKK complex (IKKβ, IKKα, and NEMO), which in turn activate IRF, participating in IFN-I transcription and IFN-I production [90]. (Table 4).

**Table 4:** The inflammatory regulation of IFN-I signaling in the NF-κB pathway

Study	Types of IFN-I	Target of action	Stimuli	Experimental models	Effect	Diseases
Sermersheim et al. 2020 [178]	IFN-β	RYR and NF-κB	MG53	MG53 knockout mice	Anti-inflammatory	Not given
Lee et al. 2021 [179]	IFN-β	PKR and NF-κB	nc886	HEp-2 cells	Anti-inflammatory	Not given
Dong et al. 2023 [180]	IFN-α	PKA/CREB/NF-κB	A2AR	PI-IBS mouse models	Pro-inflammatory	PI-IBS
Zhang et al. 2019 [181]	IFN-β	TRAF 6	miR-146a	A549 cells	Anti-inflammatory	Viral infection
Chen et al. 2023 [182]	IFN-β	TRAF 6	USP47	Mouse peritoneal macrophages	Anti-inflammatory	Viral infection

#### NF-κB

As above-mentioned, excessive activation of IFN-β has been demonstrated to lead to abnormal inflammation, tissue damage, or autoimmune disease [1]. However, inhibiting IFN-β to reduce inflammation can be achieved through suppressing NF-κB signaling. For instance, knockdown of the TRIM 72, also known as MG53, can lead to increased ryanodine receptor (RyR)-mediated intracellular calcium oscillations, further activating NF-κB signaling and inhibiting IFN-β induction, thereby suppressing the development of inflammation [178]. Conversely, nc886, a novel inhibitor of IFN-β signaling and inflammation, can restrain NF-κB signaling by suppressing protein kinase R (PKR), thus limiting excessive activation of IFN-β signaling and reducing the inflammatory state [179]. With regard to pro-inflammatory IFN-α downstream of the NF-κB signaling pathway, the Adenosine 2A receptor (A2AR) is primarily transduced through the Protein Kinase A (PKA)/CREB/NF-κB signaling pathway, which increases the level of IFN-α, promotes the viability of T cells and upregulates the secretion of inflammatory factors [180].

#### TRAF

The TNF receptor-associated factor (TRAF) proteins function as adapter, which transduce activated signals to major signaling pathways and are recruited to activate NF-κB signaling. This process is involved in inducing IFN-I signaling and modulating inflammatory cascades [183]. Recent studies have shown that downregulation of miR-146a inhibits IAV replication by enhancing IFN-β responses in vitro and in vivo through its target gene TRAF6, thereby alleviating infection-induced inflammation [181]. Similarly, USP47, a novel negative immune system regulator, also has been found to display an anti-inflammatory role via targeting TRAF. However, unlike miR-146a: USP47 removes K63-linked polyubiquitin from TRAF, thereby attenuating Sendai virus-induced IFN-β signaling conduction and inhibiting inflammation [182].

### PI3K/AKT pathway

The PI3K/AKT pathway is implicated in various human inflammatory and metabolic diseases [184]. This pathway can be induced by IFN-Is via a STAT-independent pathway [185, 186]. In response to IFN-I, the PI3K/AKT pathway displays an important role in mediating gene transcription. IFN-Is cause phosphorylation of insulin receptor substrate 1 (IRS1), which subsequently binds to subunit of PI3K-p85, thus activating PI3K's catalytic subunit p110. Consequently, inflammatory gene transcription is facilitated via phosphorylating protein kinase C (PKC) [24]. Additionally, the PI3K/ AKT signaling cascade dominates the activation of the mammalian target of rapamycin (mTOR), a critical protein mediating mRNA translation, independent of STAT family members [187, 188]. Following IFN-α and IFN-β stimulation, the mTOR pathway kinase-p70 S6K is rapidly phosphorylated and activated, which subsequently inactivates the relative repressor to increase IFN-induced mRNA translation, leading to the development of inflammation[189] (Table 5).

**Table 5:** The inflammatory regulation of IFN-I signaling in the PI3K/AKT pathway

Study	Types of IFN-I	Target of action	Stimuli	Experimental models	Effect	Diseases
Guiducci et al. 2008 [190]	IFN-α and IFN-β	PI3K	PI3K inhibitor	Primary human pDCs	Pro-and anti-inflammatory	Not given
Ding et al. 2021 [191]	IFN-α	IFN-α	IFN-α-NA	pDCs from C57BL/6 mice	Anti-inflammatory	SLE
Gairhe et al. 2021 [192]	IFN-α and IFN-β	AKT	CAV1 gene loss	Fibroblasts and serum from PAH patients, and mice	Pro-inflammatory	PAH
Matsumoto et al. 2009 [193]	IFN-α	mTOR	Rapa and AKT inhibitor	Human hepatocyte cells	Anti-inflammatory	Hepatitis C

It has been demonstrated that the production of IFN-I in pDCs is dependent on TLR [194, 195] and IRF 7 signal cascades, which modulate the inflammatory state in the physiological process [196, 197]. Guiducci et al. reported that in activated human pDCs, TLR‐mediated IRF 7 nuclear translocation regulates IFN-I, which is controlled by PI3K. This suggests that the production of IFN-I from pDCs relies on PI3K and highlights the potential therapeutic role of PI3K in autoimmune inflammation [190]. A subsequent study has furhter clarified the role of IFN-I in inhibiting inflammation through PI3K. In activated pDCs, inactivation or blockade of PI3K could neutralize IFN-α, inhibiting chemokine cytokines, and leading to the suppression of inflammation in SLE [191].

With regard to AKT, silencing Caveolin-1 (CAV1) could promote AKT-activated IFN to drive inflammatory signaling, inducing downstream IFN-α and IFN-β inflammatory responses [192]. Besides, in the human hepatocyte cells treated with rapamycin (rapa) and AKT inhibitor, it was found that mTOR signaling, rather than AKT signaling, could enhance the antiviral effects of IFN-α against the hepatitis C virus (HCV), contributing to the suppression of relative inflammation [193].

### MAPK pathway

MAPK, specially p38 and extracellular signal-regulated kinases (ERK), also play a significant role in IFN-I-modulated gene expression [24]. As reported, the suppression of p38 activity can impede IFNα-induced transcriptional activation of genes through ISRE. This inhibition, however, is not dependent on the phosphorylation of STAT1 or STAT2, nor on the formation of ISGF3 and GAS [198]. Therefore, kinase p38 is essential for IFN-I to mediate relative signaling that is independent of STATs activity, thereby modulating the inflammatory process [199, 200]. In addition to p38, ERK1/2 signaling can also be stimulated by IFN-I [201] and induced by the virus, which further produces IFN-I and facilitates inflammatory signaling [202] (Table 6).

**Table 6:** The inflammatory regulation of IFN-I signaling in the MAPK pathway

Study	Types of IFN-I	Target of action	Stimuli	Experimental models	Effect	Diseases
Wang et al. 2023 [203]	IFN-β	p38 and ERK	ZIKV	hBMECs and C57BL/6 mice	Pro-inflammatory	Viral infection
Wang et al. 2004 [202]	IFN-α and IFN-β	ERK	Myxoma virus	Mouse embryo fibroblasts	Anti-inflammatory	Viral infection

When considering viral infection in MAPK pathway, it is observed that the Zika virus (ZIKV) induces ISG expressing, which subsequently increases the levels of p38 and ERK ½, promoting the secretion of chemokines that facilitate the development of viral infection and inflammation. Meanwhile, ZIKV inhibits the phosphorylation of ribosomal protein S6 (RPS6), leading to reduced IFN-β translation and a consequent increase in of inflammation levels [203]. In addition, it is noteworthy that the myxoma virus could specifically activate ERK1/2 signaling, thereby promoting the increased secretion of IFN-α and IFN-β that resist viral infection and expansion of inflammation [202].

## Dysregulation of type I IFN signaling and inflammatory disease

Dysregulated IFN-Is signaling has been implicated in the pathogenesis of various inflammatory diseases, including autoimmune diseases, chronic infections and cancer [4–6]. In this context, we mainly focus on dysregulation of IFN-Is in autoimmune diseases and chronic inflammatory diseases.

### Functions of type I interferon in autoimmune diseases

#### Systemic lupus erythematosus (SLE)

SLE is a complex multi-system autoimmune disease and characterized by multiple organ damage [204]. Genetic variants in the IFN-I pathway and regulation of innate immune responses are also important factors in SLE susceptibility [205]. Viruses such as Epstein-Barr virus (EBV) or self-derived nucleic acids, can initiate IFN-I production via activation of intracellular receptors TLR7 and TLR9 [206]. This abnormal production of IFN-I can promote the differentiation of B cells to plasma blasts, leading to inflammation and tissue damage [207, 208]. Additionally, neutrophils may also contribute to the perpetuation of the immune response in SLE through the release of neutrophil extracellular traps (NETs), which could activate pDCs to secrete IFN-Is [209].

#### Rheumatoid Arthritis (RA)

RA is a chronic autoimmune disorder that can rapidly erode the joint cartilage and bone, leading to joint pain, stiffness, and deformities [210]. Patients with RA have been found to exhibit high levels of IFN-I. even prior to the onset of symptoms [211]. Furthermore, elevated IFN-I signatures have been shown to predict the development of RA in individuals at risk [212]. IRF 5, STAT4, and PTPN22 are the genes that have been identified as increasing the susceptibility to RA, of which are linked to the IFN-Is signaling pathway [213]. Despite the implication of IFN-Is in the pathogenesis of RA, intra-articular injections with IFN-α and intraperitoneal injection with IFN-β have been shown to prevent the occurrence or development of RA in wild-type mice or RA mouse models. This is possibly due to the ability of IFN-Is to inhibit neutrophils recruitment and activation, thereby reducing the release ROS and proteases [214].

#### Systemic Sclerosis (SSc)

SSc is a condition characterized by fibrosis, dysfunction of internal organs and a vasculopathy [215]. Reports have shown that SSc can occur in patients who have been treated with IFN-α or IFN-β for chronic myelogenous leukemia and hepatitis C [216]. IFN-I signatures have been found in the peripheral blood and affected skin of SSc patients, even in the early stages of the disease [217]. The level of IFN-Is is also linked to severe symptoms in the skin, lung, and skeletal muscle of SSc patients [218]. Additionally, higher IFN-I signatures have been found to be positively correlated with the presence of anti-topoisomerase or anti-U1-RNP antibodies in SSc patients. Conversely, a negative correlation has been observed between higher IFN-I signatures and the presence of anti-RNA polymerase III antibodies in SSc patients [216, 219].

#### Juvenile dermatomyositis (JDM)

JDM is identified by proximal muscle weakness and characteristic skin rashes. Patients with JDM have overexpressed IFN-I inducible transcripts and activated IFN-I signatures [220]. Similarly, JDM patients have increased serum IFN-α activity, which is associated with high serum muscle-derived enzymes [221]. Furthermore, the expression of IFN-I-inducible genes in muscle biopsy and the levels of proteins induced by IFN-Is, such as myxovirus resistance protein A (MxA), were found to be elevated in JDM patients. This increase in IFN-I signaling may affect both the muscle and skin tissues [222]. Moreover, the activation of TLR7 and IFN-α might lead to the expansion of immature transitional B cell population and skew the cells toward a pro-inflammatory phenotype to promote JDM progression [223].

### Functions of type I interferon in chronic infection

#### Virus infection

IFN-Is have both beneficial and detrimental effects in responding viral infection. On the beneficial side, IFN-Is can protect the host against bacterial assaults. Initial studies found that mice lacking the IFN-I receptor (IFNAR1) displayed susceptibility to various viruses, such as vesicular stomatitis virus, Semliki Forest virus, vaccinia virus and lymphocytic choriomeningitis virus (LCMV) [224, 225]. Moreover, IFN-Is possess the ability to stimulate the production of numerous antiviral proteins, including MX1, PKR, 2′-5′-oligoadenylate synthetase, IFN-induced transmembrane proteins (IFITMs), apolipoprotein B mRNA-editing enzyme catalytic polypeptide 1, and members of the TRIM family, all of which play crucial roles in inhibiting viral replication and promoting viral clearance [226, 227].

Despite the extensive antiviral effects of IFN-I, there are critical considerations. Even though IFN-I signaling can enhance the susceptibility of virally infected cells to apoptosis, thereby controlling viral replication [228], it could also lead to the death of vital cells. In vitro studies have shown that HIV can cause IFN-I-mediated upregulation of TNF-related apoptosis-inducing ligand (TRAIL) expression by pDCs, enabling these cells to induce TRAIL-dependent CD4 + T cell and B cell apoptosis. Nonetheless, by disrupting TRAIL signaling, both T and B cell functions can be restored, including the overall antibody responses to against HIV [229, 230].

However, both excessive anti-inflammation and hyper-inflammation can also lead to the detrimental effect of IFN-I on disease progression. The suppressive effect of IFN-I might contribute to chronic viral infections. Studies have shown that blocking IFN-I signaling, either through the administration of antibodies or receptor deficiency, can enhance the control of chronic infection with LCMV clone 13, mediated by CD4 + T cells [231]. IFN-Is have also been observed to dampen T cell responses by promoting the expression of immunosuppressive genes such as IL-10 and programmed cell death 1 ligand 1 (PDL1) to facilitate persistent virus infection [232]. Meanwhile, intense inflammation can result in excessive inflammation and considerable tissue damage. IFN-Is have the capacity to disrupt the TNF-induced 'cross-tolerance' which protects mice from lethal effects of endotoxins in a living body [233]. IFN-Is can effectively dismantle this TNF-induced cross-tolerance by priming chromatin, thereby facilitating robust transcriptional responses even to weak signals. This process can lead to hyperinflammation through a feedforward mechanism [234]. Moreover, IFN-I has been strongly correlated with the progression of COVID-19. The severity of COVID-19 is often accompanied by IFN-Is response, in addition to the TNF/IL-1β response, indicating that the IFN-I response could aggravate the hyper-inflammatory response by strengthening TNF/IL-1β-driven inflammation, thus influencing the severe progression of COVID-19 [235].

#### Bacterial infection

IFN-Is exhibits multifaceted effects not only in viral infections but also during bacterial infections. It serves a critical function in adjusting the host's immune response by releasing cytokines such as indoleamine 2,3-dioxygenase, inducible nitric oxide synthase (iNOS), immunoresponsive genes, and guanylate-binding proteins. The primary mechanism is through IFNγ, a part of the type II IFN family, which is indispensable for combating mycobacteria and other intracellular pathogens [236, 237]. However, the effects of IFN-Is are dual-faced, potentially assisting or hindering the host's response to bacterial infections.

IFN-Is are usually needed at the start of bacterial infections. The low level of IFN-Is helps to initiate immune response and protect against the infection. They can inhibit bacterial growth and protect human and mouse cells by depleting l-tryptophan, an essential amino acid needed by bacteria for survival [236]. Furthermore, IFN-Is might protect against Chlamydia pneumoniae infection by working in tandem with IFNγ to suppress bacterial survival [238]. IFN-Is also play a crucial role in inhibiting the replication of L. pneumophila, a common cause of pneumonia in macrophages. They activate macrophages to inhibit bacterial proliferation through reactive oxygen and reactive nitrogen [239]. Moreover, they contribute significantly to recruit protective phagocytic cells and producing chemokines like CXCL10, thereby restoring neutrophil recruitment and facilitating improved bacterial clearance [240].

Although IFN-Is can play a protective role against bacterial infections, they also have detrimental effects, particularly at high concentrations. Overwhelming levels of IFN-Is may inhibit the responsiveness of macrophages to IFNγ activation and stimulate the production of immunosuppressive molecules, potentially decreasing the immune defenses [5]. During infections with L. monocytogenes, macrophage activation by T cell- or NK cell-derived IFNγ is critical for the induction of antimicrobial pathways and elimination of intracellular bacteria [241]. While IFN-Is can significantly inhibit the responsiveness of macrophages to IFNγ, which can be attributed to the downregulation of IFNγ receptor expression on macrophages [242]. And this downregulation occurs due to the silencing of new Ifngr1 transcription by inhibitory transcriptional regulator [243]. Furthermore, during Mycobacterium leprae infections, IFN-Is can hinder macrophages from increasing the production of vitamin D-dependent antimycobacterial peptides and induce IL-10 to cause immunosuppression, which might contribute to the progression of mycobacterial diseases and result in subsequent tissue damage [244].

Additionally, IFN-Is have a detrimental impact by triggering excessive or inappropriate cell apoptosis, which can lead to the loss of essential cells and potentially intensify the severity of infections [245]. For instance, during Listeria monocytogenes infection, IFN-Is can sensitize lymphoid cells to result in large-scale apoptosis of these cells [246]. In infections caused by Tropheryma whipplei, the bacterium responsible for Whipple's disease, IFN-Is might promote macrophage apoptosis and divert macrophages to an alternatively polarized state that is more permissive to the bacteria [247]. IFN-Is can also mediate NLRP3 inflammation during gram-negative bacterial infection by the activation of caspase-11, leading to the production of proinflammatory cytokines IL-1β and IL-18, and inducing cell pyroptosis [248].

## Perspectives and conclusion

This review summarizes recent evidence indicating that IFN-Is modulate inflammation via affecting specific key factors in various signaling pathways such as JAK/ STAT pathway, TLRs pathway, NF-κB pathway, PI3K/AKT pathway, and MAPK pathway. IFN-Is' targets have the potential to become a valid approach for future interventions in inflammatory diseases, with implications for the prevention and treatment of abnormal inflammation.

However, some hurdles hamper the therapeutic use of IFN-Is at this phase, mainly due to insufficient understanding of IFN-Is mechanism, lack of sufficient animal experiments and clinical trial evidence, and the difficulties in controlling the precise inflammatory regulation [249, 250]. Further investigation is needed to elucidate the IFN-Is regulatory network specific to the progress of inflammatory diseases. As summarized, the role of IFN-Is in distinct pathways may be influenced by distinct microenvironments, and even in the same cell type, the same IFN-Is signaling may vary according to additional regulation under different conditions, adding difficulty in applying miRNA-based therapeutic approaches in the clinic. It is significant to identify the positive and negative aspects of IFN-Is-regulated signaling, exploit the IFN-Is related pathway to cure persistent inflammatory diseases, and minimize toxicity as well as side effects [89, 251]. Additionally, the main differences of various signaling pathways mediated by IFN-Is in regulating the progression of inflammatory diseases also need to be further clarified, contributing to proposing precision therapies in the future [252]. It is also notable to study whether the related limiting factors and immune activation of all ISGs could achieve balance in the IFN-Is inflammatory regulation, which may improve clinical results such as the treatment of HIV [252, 253]. Moreover, further animal experiments as well as clinical studies need to be carried out [254]. For instance, the use of JAK inhibitors in JDM still needs a large number of clinical trials to solve the existing safety and efficacy issues, even though JAK inhibitors that include Baricitinib, Tofacitinib, and Ruxolitinib have displayed preliminary efficacy of refractory juvenile JDM in several clinical cases and animal experiments [47, 255].

With regards to the development direction of IFN-Is therapy into clinics in the future, it is prospective for inflammatory diseases including new virus infections to obtain ideal IFN-Is-based therapeutic methods [14, 256, 257]. In response to the COVID-19 pandemic, research by Hoagland et al. proposed the utilization of antiviral IFN-Is system as the first line of defense against the pathogenicity of SARS-CoV-2 and supports the application of intranasal IFN-I as an early treatment effective method [258]. Nevertheless, the accurate effect of IFN-Is intervention still needs to be determined. To be specific, timely and potent IFN-Is production (18–24 h post-infection) promotes both innate and acquired immune responses, whereas delayed IFN-Is production (3–4 days post-infection) actually contributes to ineffective anti-infection as well as excessive inflammation [9]. Besides, IFN-Is intervention may have a more pronounced effect on organisms genetically modified to lack innate immune sensors, like TLRs [258]. A complete evaluation of the immune-inflammatory response of IFN-Is against SARS-CoV-2 is crucial for designing harmless and effective vaccines in clinical treatment. In addition, it is necessary to determine the role of IFN-Is treatment in late disease and lethal models to further delineate the nuances of boosting IFN before, during, and/or after SARS-CoV-2 infection [258].

## Data Availability

All data relevant to this review is included in the text, references, and figures.

## Abbreviations

LPS: Lipopolysaccharide
TRIF: Toll-receptor-domain-containing adapter-inducing interferon-β
SLE: Systemic lupus erythematosus
JDM: Juvenile dermatomyositis
SSc: Stemic sclerosis
MyD88: Myeloid differentiation primary response gene 88
RIG-I: Retinoic acid-inducible gene I
TLRs: Toll-like receptors
PRRs: Pattern recognition receptors
DEXD/H box: DEAD and DEAH box
DAI: DNA-dependent activator of IFN-regulatory factors
MDA5: Melanoma differentiation-associated gene 5
NF-κB: Nuclear factor-κB
MAPK: Mitogen-activated protein kinases
AKT: Serine-threonine kinase
PI3K: Phosphoinositide 3-kinase
UVB: Ultraviolet B
RSV: Respiratory syncytial virus
MO-IFN: IFN-I signaling-dependent monocyte subpopulation
JAK: Janus kinase
GILZ: Glucocorticoid-induced leucine zipper
CIA: Collagen II-induced arthritis
IAV: Influenza A virus
SHP2: Src homology 2-containing protein tyrosine phosphatase 2
CBP: Co-activator cAMP-response element binding protein (CREB)-binding protein
GTD: Gastrodin
CBD: Cannabidiol
Cap: Capsid protein
dsDNA: Double-stranded DNA
ATP: Adenosine 5'-tri Phosphate
GTP: Guanosine 5'-triphosphate
CDNs: Cyclic dinucleotides
ER: Endoplasmic reticulum
mtDNA: Mitochondrial DNA
TNF: Tumor necrosis factor
NLRP3: Nucleotide-binding oligomerization domain, leucine-rich repeat and pyrin domain-containing 3
Polβ: Polymerase β
RRBE: Red rice bran extract
ILC2: Type 2 innate lymphoid cells
AIP: Aryl hydrocarbon receptor-interacting protein
PACT: Protein activator of the interferon-induced protein kinase
PRKRA: Protein kinase, interferon-inducible double-stranded RNA-dependent activator
SARS-CoV-2: Severe acute respiratory syndrome coronavirus 2
CVB3: Coxsackievirus B3
LAB: Lactic acid bacteria Lactobacillus
mtRNA: Mitochondrial RNA
RNF114: RING finger protein 114
COVID-19: Coronavirus disease 2019
RyR: Ryanodine receptor
PKR: Protein kinase R
A2AR: Adenosine 2A receptor
PKA: Protein Kinase A
TRAF: TNF receptor-associated factor
IRS1: Insulin receptor substrate 1
PKC: Phosphorylating protein kinase C
mTOR: Mammalian target of rapamycin
CAV1: Caveolin-1
rapa: Rapamycin
HCV: Hepatitis C virus
ERK: Extracellular signal-regulated kinases
ZIKV: Zika virus
RPS6: Ribosomal protein S6
EBV: Epstein-Barr virus
NETs: Neutrophil extracellular traps
MxA: Myxovirus resistance protein A
CARD: Caspase activation and recruitment domain
LCMV: Lymphocytic choriomeningitis virus
TRAIL: TNF-related apoptosis-inducing ligand
IPS-1: IFN-Is promoter stimulator-1
RLRs: RIG-I-like receptors
IFI6: IFN-α-inducible protein 6
PCV3: Porcine circovirus 3
HIV: Human immunodeficiency virus
CQ-FM: Chloroquine loaded by filamentous micelles
DAMP: Damage-associated molecular patterns
IKK: Inhibitor of NF-κB (IκB) kinase
MAVS: Mitochondrial antiviral signaling protein
DCs: Dendritic cells
IRF: Interferon regulatory factor
TBK1: Activation of tank-binding kinase-1
PDL1: Programmed cell death 1 ligand 1
iNOS: Inducible nitric oxide synthase
IFITMs: IFN-induced transmembrane proteins
GAS: Gamma-activating sequence
STING: Stimulator of IFN genes
TYK2: Tyrosine kinase 2
ISG: IFN-stimulated genes
ISRE: IFN-stimulated response elements
cGAMP: Circular GAMP
cGAS: Cyclic GMP-AMP synthase
IFNAR: Interferon alpha receptor
STING: Stimulator of interferon genes
Lpr: Lymphoproliferation
BHK-21 cells: Baby hamster Syrian kidney cells
Ab: Antibody
PAM: Porcine alveolar macrophages
PAMPs: Pathogen-associated molecular patterns
BALF: Bronchoalveolar lavage fluid
MRSA: Methicillin-resistant Staphylococcus aureus
NRF2: Nuclear factor erythroid 2-related factor 2
HS: Hemorrhagic shock
RA: Rheumatoid arthritis
PON1: Paraoxonase-1
CHIKV: Chikungunya virus
CCL11: C–C Motif Chemokine Ligand 11
TRIM25: Tripartite motif protein 25
Nsp9: Nonstructural protein 9
PRRSV: Syndrome virus
DENV: Dengue virus
T1DM: Type 1 diabetes mellitus
ARDS: Acute respiratory distress syndrome
ALX/FPR2: Formyl peptide receptor 2
PBMCs: Peripheral blood mononuclear cells
BMDM: Bone marrow derived macrophages
MCT: Mast cell tryptase
CCI: Controlled cortical impact
TBI: Traumatic brain injury
MEFs: Mouse embryonic fibroblasts
RIPK1: Receptor-interacting serine/threonine-protein kinase 1
CDT: Cytolethal distending toxi
A. cinnamomea: Antrodia camphorata
s.Typhimurium: Serovar Typhimurium
MCPIP-1: Monocyte chemotactic protein-induced protein 1
NEMO: NF-Kappa-B Essential Modulator
PI-IBS: Post-infectious irritable bowel syndrome
PAH: Pulmonary arterial hypertension
hBMECs: Human brain microvascular endothelial cell
